# Gene Profiling of Aortic Valve Interstitial Cells under Elevated Pressure Conditions: Modulation of Inflammatory Gene Networks

**DOI:** 10.4061/2011/176412

**Published:** 2011-08-18

**Authors:** James N. Warnock, Bindu Nanduri, Carol A. Pregonero Gamez, Juliet Tang, Daniel Koback, William M. Muir, Shane C. Burgess

**Affiliations:** ^1^Department of Agricultural and Biological Engineering, Mississippi State University, Mississippi State, MS 39762, USA; ^2^Life Science and Biotechnology Institute, Mississippi State University, Mississippi State, MS 39762, USA; ^3^Department of Basic Sciences, Mississippi State University, Mississippi State, MS 39762, USA; ^4^Department of Animal Sciences, Purdue University, West Lafayette, IN 47907, USA

## Abstract

The study aimed to identify mechanosensitive pathways and gene networks that are stimulated by elevated cyclic pressure in aortic valve interstitial cells (VICs) and lead to detrimental tissue remodeling and/or pathogenesis. Porcine aortic valve leaflets were exposed to cyclic pressures of 80 or 120 mmHg, corresponding to diastolic transvalvular pressure in normal and hypertensive conditions, respectively. Linear, two-cycle amplification of total RNA, followed by microarray was performed for transcriptome analysis (with qRT-PCR validation). A combination of systems biology modeling and pathway analysis identified novel genes and molecular mechanisms underlying the biological response of VICs to elevated pressure. 56 gene transcripts related to inflammatory response mechanisms were differentially expressed. TNF-**α**, IL-1**α**, and IL-1**β** were key cytokines identified from the gene network model. Also of interest was the discovery that pentraxin 3 (PTX3) was significantly upregulated under elevated pressure conditions (41-fold change). In conclusion, a gene network model showing differentially expressed inflammatory genes and their interactions in VICs exposed to elevated pressure has been developed. This system overview has detected key molecules that could be targeted for pharmacotherapy of aortic stenosis in hypertensive patients.

## 1. Introduction

The pathogenesis of aortic stenosis (AS) is a largely understudied research area, compared to other cardiovascular diseases, which has major human health implications. Historically, AS has been considered an age-related, passive, degenerative disease. However, during the past 15 years, indisputable evidence has shown that AS is an active, cell-mediated process. Nonrheumatic AS is characterized by chronic inflammation, increased extracellular matrix (ECM) remodeling, proliferation, and differentiation of valvular interstitial cells (VICs) and the development of calcific lesions on the valve [1, 2]. Nonrheumatic AS is preceded by aortic sclerosis, a condition of valve thickening in which left ventricular outflow is not obstructed. Aortic sclerosis is associated with a 50% increase in death from all cardiovascular causes and increases the risk of myocardial infarction, heart failure, and stroke [3]. Progressive AS, in which obstruction to left ventricle outflow is present, produces left ventricular hypertrophy, left ventricular diastolic and systolic dysfunction, congestive heart failure, angina, arrhythmias, and syncope [4]. Severe symptomatic AS is associated with a life expectancy of less than 5 years [5]. In 2009, AS was directly responsible for over 13,752 American deaths and was an underlying factor in an additional 27,380 deaths and 49,000 hospital discharges. Though the disease is associated with significant clinical consequences, there is currently no effective therapy for valve disease other than surgical aortic valve replacement [6]. 

*In vivo* studies have shown that a causal link exists between hypertension and AS [7]. This is supported by numerous *in vitro* studies that have shown that elevated cyclic pressure plays an important role in valve ECM synthesis, proinflammatory and cathepsin gene expression [8–11]. In addition, it has been reported that transvalvular pressure has a direct effect on VIC stiffness and collagen synthesis [12]. The potential mechanisms connecting hypertension with initiation and progression of aortic valve disease include (1) hypertensive pressure raises the diastolic transvalvular pressure across the valve, increasing the mechanical strain experienced by the leaflets; (2) hypertension may disturb the hemodynamic environment (i.e., compression of the ECM, altered flow patterns), thus influencing valve cell behavior; and (3) hypertension may play a key role in the activation of several biological processes that induce aortic valve remodeling and disease [13]. 

We present here the first study of VIC gene expression profiling in an *ex vivo* model of elevated cyclic pressure. The data generated have enabled us to identify mechanosensitive gene networks, and we have also investigated VIC expression of a subset of genes associated with inflammation. It was hypothesized that expression of several proinflammatory genes, such as TNF-*α* and IL-6, would be significantly increased as clinical studies have shown these to colocalize with calcific regions in explanted aortic valves from prehypertensive patients [14]. TNF-*α* has also been associated with matrix remodeling through the expression of MMP-1 and -3 [15]. Additionally, TNF-*α* and other cytokines, such as IL-1*β*, express enzymes generating oxidants (O_2_^−^) capable of promoting low-density lipoprotein (LDL) oxidation [16]. Proteoglycans trap LDL in the tissue, and oxidative modification leads to endothelial expression of adhesion molecules (ICAM-1 and VCAM-1) and chemoattractants (MCP-1). 

## 2. Materials and Methods

### 2.1. Tissue Harvest

**Figure 1:**
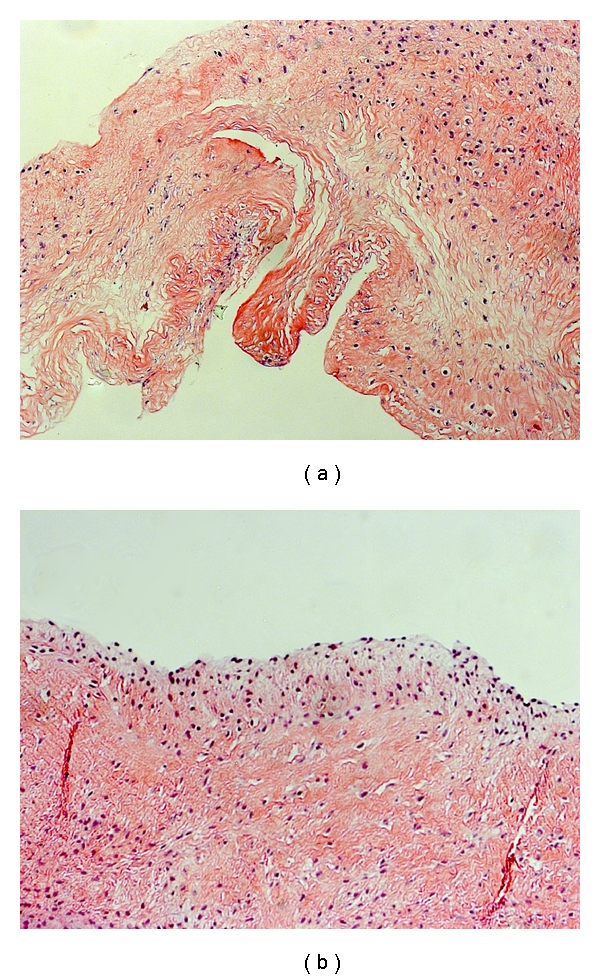
(a) H & E stain of a valve leaflet, confirming that the endothelium has been removed by 5-minute incubation with collagenase II followed by gentle swabbing. (b) Positive control sample showing an intact endothelium on a valve leaflet not treated with collagenase II and gentle swabbing. 10x magnification.

Aortic valves were collected from six individual female Yorkshire/Hampshire pigs immediately after death. Animals were less than 6 months of age with a postslaughter weight of no more than 120 lbs (Sansing Meat Services, Maben, MS). Valves were rinsed twice in ice-cold sterile Phosphate Buffer Saline (PBS, Sigma, St Louis, MO) and transported to the laboratory on ice. Leaflets were cut one third of the distance from the annulus. Valve leaflets did not show any sign of degeneration, tearing or calcification. To ensure that only valve interstitial cells (VICs) were present in each sample, the endothelial cell layer was removed from each leaflet surface by immersion of valve tissue in collagenase II (2 *μ*g/mL in serum free DMEM, Worthington Biochemical Corp.) for 5 minutes at 37°C and 5% CO_2_. The leaflet surface was then gently swabbed. Confirmation that the EC had been removed was done through Hemotoxylin and Eosin staining (Figure 1). Valve leaflets were rinsed twice with PBS to remove excess collagenase II before being incubated overnight in DMEM supplemented with 10% Fetal Bovine Serum (FBS; Hyclone, Logan UT) and 1% antibiotic/antimycotic solution (Sigma). The tissue had approximate dimensions of 12 mm × 15 mm. 

### 2.2. Pressure Studies

**Figure 2:**
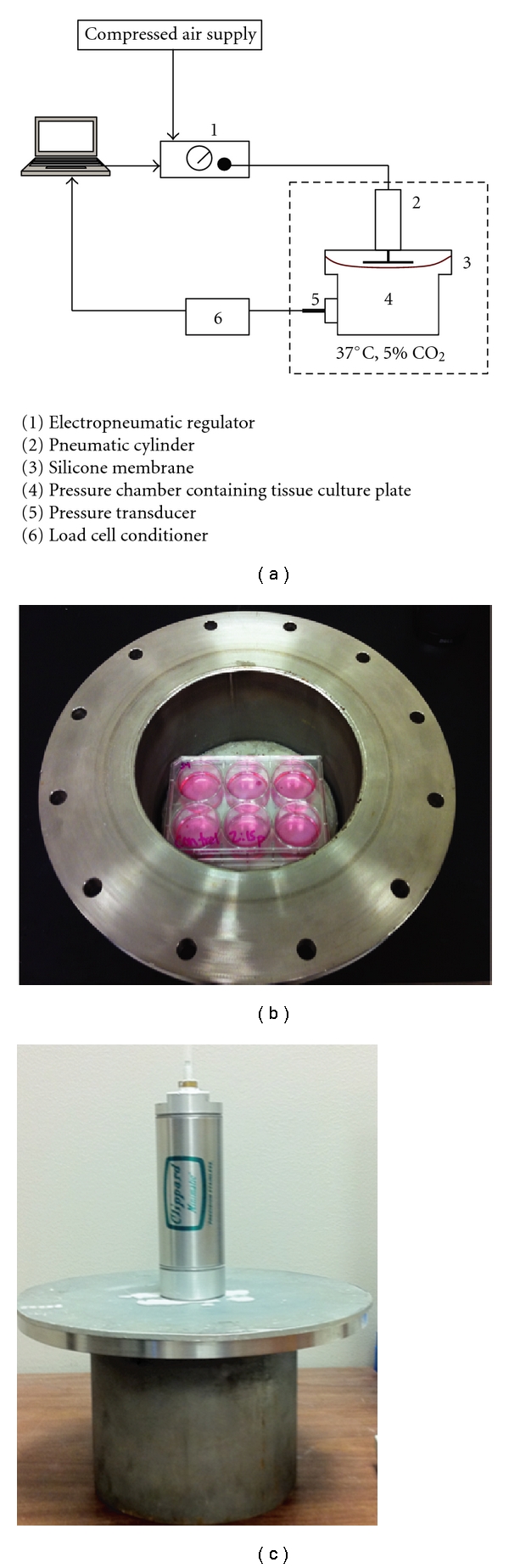
(a) Schematic diagram of the pressure system. A computer controls an electropneumatic regulator (1), controlling the flow of compressed air to a pneumatic cylinder (2) the cylinder exerts a compressive force on a silicone membrane (3) reducing the volume in the pressure chamber (4) and subsequently increasing the atmospheric pressure surrounding the leaflets that are in tissue culture plates in the chamber. A pressure transducer (Omega Engineering, Inc, PX302-200GV) (5) records the internal pressure of the chamber, which is relayed back to the computer via a load cell conditioner (Encore Electronics, Inc., Model 4025-101) (6). The voltage and the pressure signal were acquired with a data acquisition card module (Measurement Devices, PMD1608) and monitored using an in-house developed LabVIEW graphical user program (LabVIEW, National Instruments). The system was placed inside an incubator to maintain a 37°C, 5% CO_2_ humidified atmosphere. (b**)** Photograph of a six-well plate in the pressure chamber before being sealed. (c) Photograph of the sealed pressure chamber prior to being place in the 37°C incubator.

**Figure 3:**
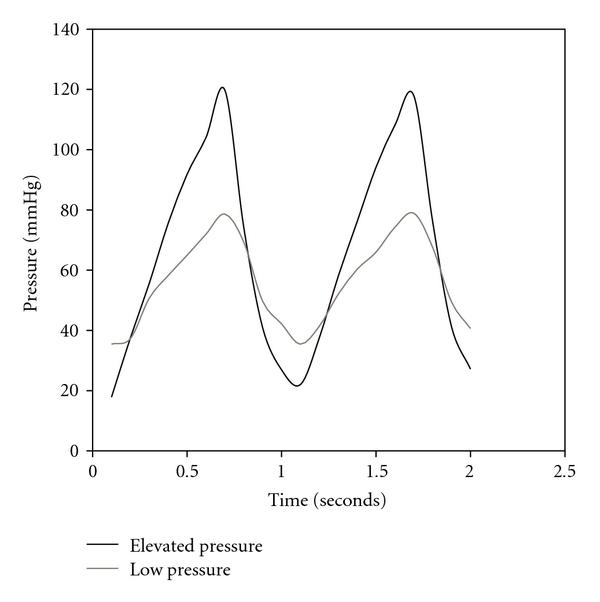
Pressure waveforms generated from the pressure system.

To investigate the effects of cyclic pressure, a custom-made, computer-operated dynamic pressure system was used. A schematic diagram and photos of the system are shown in Figure 2. Similar *ex vivo* systems have been used in the past to demonstrate changes in extracellular matrix protein synthesis and remodeling under elevated pressure conditions [8, 11]. Additionally, this system has been used to demonstrate a correlation between elevated pressure and proinflammatory gene expression in aortic valve interstitial cells [9]. Leaflet tissue was placed in a six-well tissue culture plate and immersed in 3 ml of culture medium. The tissue culture plates were placed in the pressure chamber and exposed to cyclic pressures of 80 mmHg or 120 mmHg, corresponding to diastolic transvalvular pressure in normotensive and hypertensive conditions [17], respectively, at a frequency of 1 Hz (sinusoidal wave; 0.6 sec influx, 0.4 sec outflux) for 24 hours.  Figure 3 shows representative waveforms. At low-pressure conditions, pressure cycled between 35 mmHg and 80 mmHg, with amplitude of 45 mmHg. Under elevated pressure conditions, the maximum pressure was 120 mmHg and the minimum was 25 mmHg, providing amplitude of 95 mmHg. The pressure system exposed tissues to mechanical stimulation by increasing the air pressure above the supernatant media. To produce a change in the pressure within the chamber, the pneumatic piston moved downward in the chamber space, and the silicone gasket was stretched downward, reducing the volume of the chamber, and increasing the air pressure. During experiments, the pressure chamber was placed in an incubator at 37°C. The pH of the culture medium was measured prior to and after each test using a pH meter. pH readings of culture medium ranged between 7.3–7.4, indicating no significant changes. Each experimental group contained three biological replicates.

### 2.3. Array Experiments

Upon completion of pressure experiments, each leaflet was rinsed twice with sterile PBS, submerged in 1 mL of RNA later RNA Stabilization Reagent (Qiagen) to avoid changes in RNA expression and stored at −80°C until RNA extraction. Total RNA from porcine aortic valve interstitial cells (VICs) was extracted using the RNeasy Mini kit (Qiagen, Valencia, CA) following the manufacturer's instructions and stored at −80°C. The quantity and quality of RNA was confirmed by spectrophotometry (A260/A280 ratio) and capillary electrophoresis (2100 Bioanalyzer; Agilent Technologies, Palo Alto, CA) by using an RNA 6000 Picochip kit. Target preparation for microarray analysis was performed according to the manufacturer's established protocol (www.affymetrix.com). Briefly, a total of 100 ng of RNA from each sample was used for single-stranded cDNA synthesis with SuperScript II reverse transcriptase and T7-Oligo(dT) primer/Poly(A) controls (Affymetrix, Santa Clara, CA). The single-stranded cDNA was then converted to double-stranded cDNA by using *Escherichia coli* DNA polymerase I (Affymetrix) for the first cycle. From template cDNA, biotin-labeled cRNA was prepared in an *in vitro* transcription (IVT) reaction by using the MEGAscript High-Yield Transcription Kit (Ambion Inc, Austin, TX). Following *in vitro* transcription, 600 ng of cRNA from each replicate was used for the second-cycle first-strand cDNA synthesis. The first-strand cDNA from the second cycle was then converted to second-strand cDNA. From second-strand cDNA template, biotin-labeled cRNA was prepared in an *in vitro *transcription (IVT) reaction by using GeneChip IVT Labeling Kit (Affymetrix) according to the manufacturer's instructions. Then 15 *μ*g of biotin-labeled cRNA was fragmented in 1′ fragmentation buffer solution provided with the GeneChip Sample Cleanup Module (Affymetrix) at 94°C for 35 min. A total of 10 *μ*g of fragmented biotin-labeled cRNA per replicate in hybridization mixture was then hybridized to Porcine Genome Array from Affymetrix GeneChips and incubated overnight at 45°C in a rotating hybridization oven, all according to the manufacturer's instructions (Affymetrix). After >16 h of hybridization, the mixture was removed, and, in several cycles, the chips were washed with nonstringent buffer and stained with streptavidin/phycoerythrin (SAPE) antibody solution according to the manufacturer's instructions by using an Affymetrix FS-450 fluidics station. The data were collected using Affymetrix GeneChip Scanner 3300 (Affymetrix). Three chips were used for each experimental condition and the RNA for each chip was obtained from three different leaflets.

### 2.4. Statistical Analysis of Array Data

Microarray data was analyzed using a mixture model approach as previously described [18]. Briefly, microarray analysis was performed for expression differences assuming that genes in alternative treatments are expressed or not in the following combinations: (i) not expressed in either condition, (ii) expressed only under the first condition, (iii) expressed only under the second condition, and (iv) expressed under both conditions, giving rise to 4 possible clusters with two treatments. The number of these combinations/clusters was determined by Akaike's Information Criterion (AIC) and the Bayesian Information Criterion (BIC) [19, 20]. 

### 2.5. Gene Expression Analysis by Quantitative Real-Time Polymerase Chain Reaction

To confirm the fold changes in gene expression from the array data, semi-quantitative reverse transcriptase polymerase chain reaction (qRT-PCR) was done to measure the relative change in mRNA expression. Real-Time qRT-PCR was carried out with 10 ng of total RNA using a Bio-Rad iCycler thermocycler and iScript one-step SYBR Green kit, following the manufacturer's instructions. Primer sequences (Table 1) were designed using Primer 3 software [14]. Sequences were selected that crossed intron/exon boundaries to ensure the elimination of genomic DNA. cDNA synthesis and PCR amplification were performed using the following steps: 50°C for 30 mins then the reaction mixture was heated to 95°C for 5 min; a 45 cycle two-step PCR was performed consisting of 95°C for 15 s followed by 1 min at 60°C. Following amplification, a melt curve was generated that confirmed primer specificity. Expression values for each gene were calculated relative to 18s mRNA levels. The mean fold change (*n = 5*) was calculated using the 2^(-ΔΔCt)^ method.

### 2.6. Network Modeling of Gene Expression Data

Affymetrix probe IDs that did not belong to the null distribution based on the mixture model analysis were mapped to Ensembl porcine gene accessions using Ensembl Biomart [21]. To identify the molecular functions, biological networks and signaling pathways in VICs responsive to cyclic pressure, pathway analysis using Ingenuity Pathways Analysis (IPA; Ingenuity Systems, California) was carried out as described previously [22]. Human orthologs for porcine genes (obtained from Biomart) were used in IPA. IPA generated networks that are no more than 35 genes/proteins in size. Based on the overlap between the genes in user dataset and a reference set (which is often the entire genome), IPA estimates the probability that genes in a network were found together due to chance. Networks scoring ≥ 2, with >99% confidence of not being generated by chance were considered to be significant. Annotations from scientific literature stored in the Ingenuity Pathways Knowledge Base (IPKB) were used to determine biological functions of the identified networks. Fisher exact test was used to calculate the *P*-value, the probability of each biological function/disease or pathway being assigned by chance. A *P* ≤ 0.05 (adjusted for multiple testing for statistical rigorousness) was used to select highly significant biological functions and pathways represented in the gene expression datasets. 

## 3. Results

**Figure 4:**
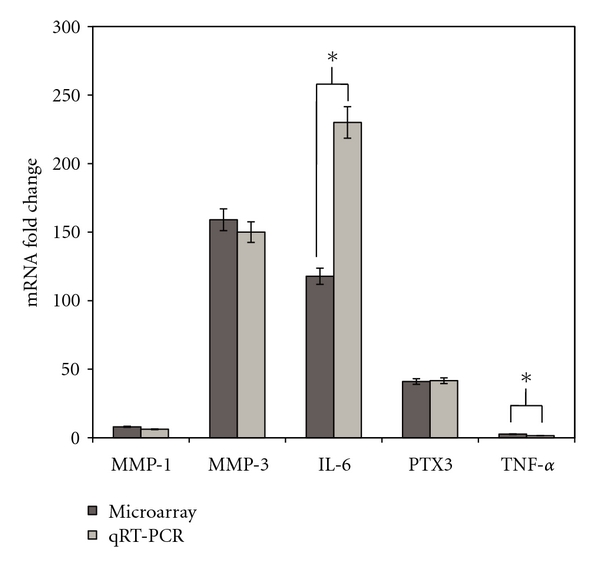
Confirmation of microarray data by real-time qRT-PCR. The fold change in gene expression was significantly higher in VICs exposed to elevated pressure compared to VICs exposed to normal cyclic pressure. *Represents a significant difference between microarray and qRT-PCR data.

**Figure 5:**
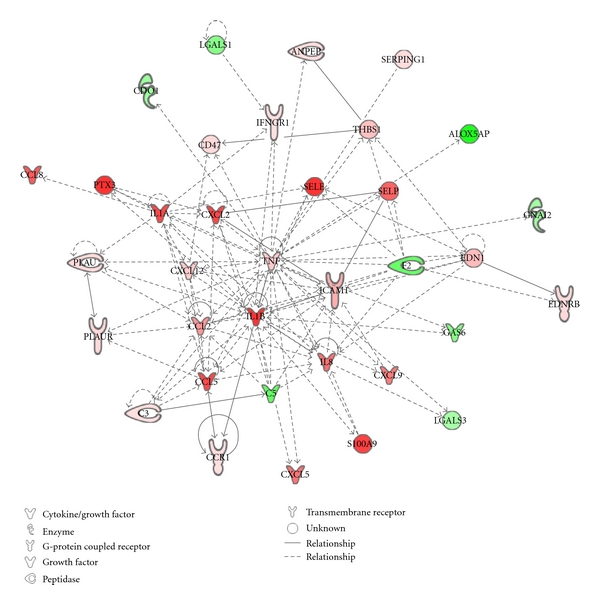
Inflammatory gene network associated with upregulation of TNF*α*. In the network, green represents genes with reduced expression while pink and red nodes denote a significant increase in gene expression.

Transcriptome analysis of VICs was performed to determine the distinct genetic profile of VICs exposed to 80 mmHg (control) and 120 mmHg cyclic pressure. The mixture model method was used as a preliminary tool to cluster genes that could be important for the biology under investigation. In this analysis, 3 clusters with 49.8% of the differences in the null cluster were found, meaning that approximately 50% of the transcriptome was impacted by treatments to some degree. However, only ~6,000 of those genes could be differentiated from the null cluster with high probability (*P* < 0.0001). The microarray data was validated at the mRNA level by qRT-PCR (Figure 4). Based on the microarray data, we chose three genes known to be mechanosensitive and associated with aortic stenosis: MMP-1, MMP-3, and IL-6. Further, MMP3 and IL6 had the highest fold change detected by microarray. qRT-PCR was also performed on PTX3 and TNF-*α*, as these were key genes identified in the network analysis. Upregulation of all five genes in response to elevated pressure found in the microarray was confirmed by qRT-PCR. There was a significant difference in the fold change of IL6 and TNF-*α*; however, microarray and qRT-PCR both showed these genes to be upregulated in the presence of elevated pressure. A pathway analysis using IPA was completed to provide the basis for determining molecular functions, pathways, and networks that were important for the VIC response to altered pressure. IPA analysis showed that 56 genes related to inflammation were differentially expressed; 35 of these genes were up regulated and 21 genes were downregulated, as shown in Tables 2 and 3, respectively. At the chosen statistical thresholds, 16 networks were identified. The network centered on tumor necrosis factor (TNF-*α*) that included molecules involved in the inflammatory response is shown in Figure 5. This network included all genes differentially expressed by elevated pressure, as shown in Tables 2 and 3.

## 4. Discussion

The limited success of antiatherosclerotic therapies and the realization that distinct differences exist between the pathogenesis of atherosclerosis and AS suggests that innovative pharmacotherapies are needed. Analysis of gene expression changes in the context of response, networks, and pathways can expedite understanding of the molecular mechanisms that govern the VIC response to pressure. In the present study, novel molecular mechanisms that are activated in VICs during exposure to elevated pressure conditions were identified. Our results show that elevated pressure induces a gene expression pattern in cells that is considerably similar to that seen in aortic valve disease [1], in terms of altered expression of ECM proteins (MMP-1, MMP-3) [23, 24] and proinflammatory cytokines (IL-1*β*, IL-6) [15, 25, 26]. These results underline the key role of hypertension as an initiating factor in the onset of aortic valve pathogenesis. Modeling these genes to identify networks has facilitated the discovery of some very specific genes that could potentially be targeted for the treatment of aortic heart valve disease.

Previous histological studies of stenotic valves show that TNF-*α* and IL-6 both colocalize with ox-LDL. These tend to localize in the fibrosa at the vicinity of calcified areas [14]. Hence, the findings from the microarray data are supported by clinical observations. Creation of the gene network model provides a systems view of the molecular mechanisms and enables us to identify how various genes interact. TNF-*α* expression was increased under elevated pressure conditions and appears to be a key molecule in this network as it promotes expression of several adhesion molecules, including E-selectin, P-selectin, and ICAM-1. Although adhesion molecules are more typically associated with endothelial cells, it is known that activated vascular smooth muscle cells can express adhesion molecules as part of the inflammatory process [27]. TNF-*α* also interacts with the cytokines IL-1*α* and IL-1*β*. Cytokine-targeting therapy that specifically targets TNF-*α*, IL-1, or IL-6 could be an effective antiinflammatory treatment for retarding disease progression. IL-1*α* and IL-1*β* promote the expression of long-chain pentraxin 3 (PTX3). Clinical studies have also shown that PTX3 levels are elevated in AS patients [28]. The short chain pentraxin, C-reactive protein, has been offered as an early diagnostic marker for cardiovascular diseases. However, because it can be produced in several organs, its reliability has been questioned. PTX3 could be a potential alternative, especially heart valve disease, and it has been proposed as a new candidate marker for acute and chronic heart diseases [29]. PTX3 is also involved in controlling inflammation and tissue remodeling and could therefore be a potential candidate for early AS therapy. Recent studies have shown that PTX3 has a nonredundant regulatory and cardioprotective role in acute myocardial infarction in mice [30]. 

The results of the present study are consistent with our global hypothesis that elevated pressure contributes to the development of aortic valve disease. However, certain limitations of the present study should be acknowledged. First, the small sample for microarray analysis may decrease the sensitivity of the study. However, the Mean-Difference-Mixture-Model (MD-MM) method used for statistical analysis is generally superior to other methods in most situations. The method is particularly advantageous in situations where there are few replicates, poor signal to noise ratios, or non-homogenous variances [18]. Second, the amount of total RNA isolated from tissue was insufficient for microarray analysis without nonlinear amplification. Therefore, a two-cycle amplification was necessary to increase the amount of cRNA for testing. Alternative methods of RNA isolation, such as the Qiagen lipid kit, may alleviate this limitation in future studies. As valve leaflets were only exposed to pressure, we did not address the potentially important role of other mechanical factors that are part of the valve mechanical environment *in vivo* such as tensile and compressive strain, shear stress, and flexure. When these forces are combined, they could potentially have an antagonistic or a synergistic effect. Another limitation in our study is our Pathway analysis using IPA. While IPA helped in identifying molecular networks responsive to elevated pressure in VICs, it failed to capture species-specific information that could be pertinent to effects of pressure on VICs. Furthermore, information within IPA knowledge base is obtained from scientific literature, and thus gene functions and interactions are subject to the last update of the software. 

To obtain a pure population of VICs, the endothelium was removed from leaflets. Endothelial denudation may have multiple effects on the biology of the tissue. It has been proposed that alterations in the mechanical environment of the leaflets could be transduced into a pathobiological response via a two-way communication system between endothelial and interstitial cells. Denudation disrupts this communication and may expose the subendothelial interstitial cells to mechanical stimuli that they do not see when the endothelium is intact. This limitation would also be present in cell culture; however, by using an organ culture system, VICs were retained in their native three-dimensional ECM. The ECM is important for the transmission of mechanical signals to cells and thus, this system has a distinct advantage over mechanical studies performed with isolated cells. The alternative is *in vivo* studies. Although these have greater physiological relevance, they do not allow for strict mechanical characterization or isolation of the effects of pressure.

In conclusion, pressure-induced changes in the porcine aortic valve interstitial cell transcriptome are reported. This study provides rationale for further investigation of highly connected and highly regulated genes as potential therapeutic targets.

## Figures and Tables

**Table 1:** Primer sequences used for qRT-PCR.

Gene	GenBank accession no.	Primer sequence	Size (bp)
MMP-3	NM_001166308.1	5′-TGTGGAGTTCCTGATGTTGG-3′(F) 5′-GGCTGAAGTCTCCGTGTTCT-3′(R)	240
MMP-1	NM_001166229	5′-TTTCCTGGGATTGGCAAC-3′(F) 5′-TCCTGCAGTTGAACCAGCTA-3′(R)	233
IL-6	NM_214399.1	5′-CACCAGGAACGAAAGAGAGC-3′(F) 5′-GTTTTGTCCGGAGAGGTGAA-3′(R)	204
PTX3	NM_002852.3	5′-GGGACAAGCTCTTCATCATGCT-3′(F) 5′-GTCGTCCGTGGCTTGCA-3′(R)	71
TNF-*α*	NM_214022.1	5′-GAAGACACACCCCCGAACAGGCA-3′(F) 5′-ACGTGGGCGACGGGCTTATCT-3′(R)	379

**Table 2:** Upregulated genes related to inflammation. All genes had a *P* ≤ 0.001.

Ensembl Gene ID	Gene name	Description	Fold change
ENSSSCG00000014986	MMP3	Matrix metallopeptidase 3 (stromelysin 1, progelatinase)	159.845
ENSSSCG00000015385	IL6	Interleukin 6 (interferon, beta 2)	117.817
ENSSSCG00000006286	SELE	Selectin E	74.336
ENSSSCG00000011727	PTX3	Pentraxin 3, long	41.052
ENSSSCG00000008087	IL1B	Interleukin 1, beta	20.185
ENSSSCG00000017482	CSF3	Granulocyte colony-stimulating factor Precursor (G-CSF)	15.267
ENSSSCG00000006588	S100A9	S100 calcium binding protein A9	9.664
ENSSSCG00000008959	CXCL2	CXCL2	9.498
ENSSSCG00000008090	IL1A	Interleukin 1, alpha	9.361
ENSSSCG00000017705	CCL5	Chemokine (C-C motif) ligand 5	9.339
ENSSSCG00000017721	CCL8	Chemokine (C-C motif) ligand 8	8.386
ENSSSCG00000006288	SELP	Selectin P (granule membrane protein 140 kDa, antigen CD62)	8.002
ENSSSCG00000014985	MMP1	Matrix metallopeptidase 1 (interstitial collagenase)	7.976
ENSSSCG00000008953	IL8	Interleukin 8	7.279
ENSSSCG00000008957	CXCL5	Alveolar macrophage chemotactic factor 2 Precursor (Alveolar macrophage chemotactic factor II) (AMCF-II)	7.189
ENSSSCG00000008975	CXCL9	Chemokine (C-X-C motif) ligand 9	6.687
ENSSSCG00000017723	CCL2	Chemokine (C-C motif) ligand 2	5.243
ENSSSCG00000012853	IRF7	Interferon regulatory factor 7	4.625
ENSSSCG00000013655	ICAM1	Intercellular adhesion molecule 1	4.041
ENSSSCG00000001050	EDN1	Endothelin 1	3.383
ENSSSCG00000004789	THBS1	Thrombospondin 1	3.113
ENSSSCG00000001404	TNF	Tumor necrosis factor Precursor (TNF-alpha)(Tumor necrosis factor Ligand superfamily member 2)(TNF-a)(Cachectin)	2.626
ENSSSCG00000010414	CXCL12	Chemokine (C-X-C motif) ligand 12	2.62
ENSSSCG00000000708	TNFRSF1A	Tumor necrosis factor receptor superfamily, member 1A	2.327
ENSSSCG00000009002	TLR2	Toll-like receptor 2	2.157
ENSSSCG00000003065	PLAUR	Plasminogen activator, urokinase receptor	1.937
ENSSSCG00000009477	EDNRB	Endothelin B receptor Precursor (ET-B)(Endothelin receptor nonselective type)	1.859
ENSSSCG00000011942	CD47	CD47 molecule	1.858
ENSSSCG00000001346	ABCF1	ATP-binding cassette subfamily F member 1	1.786
ENSSSCG00000010312	PLAU	Plasminogen activator, urokinase	1.747
ENSSSCG00000013181	SERPING1	Serpin peptidase inhibitor, clade G (C1 inhibitor), member 1	1.71
ENSSSCG00000007440	CD40	CD40 molecule, TNF receptor superfamily member 5	1.674
ENSSSCG00000004156	IFNGR1	Interferon gamma receptor 1	1.6
ENSSSCG00000001849	ANPEP	Alanyl (membrane) aminopeptidase	1.547
ENSSSCG00000011322	CCR1	Chemokine (C-C motif) receptor 1	1.542
ENSSSCG00000000244	PPBP	Platelet basic protein Precursor (PBP)(C-X-C motif chemokine 7)(Small-inducible cytokine B7)	1.533
ENSSSCG00000013551	C3	Complement component 3	1.514

**Table 3:** Down regulated genes related to inflammation. All genes had a *P* ≤ 0.001.

Ensembl gene ID	Gene name	Description	Fold change
ENSSSCG00000005055	LGALS3	Lectin, galactoside-binding, soluble, 3	−1.575
ENSSSCG00000011993	PROS1	Protein S (alpha)	−1.654
ENSSSCG00000011399	GNAI2	Guanine nucleotide binding protein (G protein), alpha inhibiting activity polypeptide 2	−1.69
ENSSSCG00000001408	AIF1	Allograft inflammatory factor 1 (AIF-1)(Ionized calcium-binding adapter molecule 1)(Protein G1)	−1.715
ENSSSCG00000014310	CXCL14	Chemokine (C-X-C motif) ligand 14	−1.73
ENSSSCG00000013030	PRDX5	Peroxiredoxin 5	−1.751
ENSSSCG00000014219	CDO1	Cysteine dioxygenase, type I	−1.791
ENSSSCG00000012840	CD151	CD151 molecule (Raph blood group)	−1.851
ENSSSCG00000015997	PRKRA	Protein kinase, interferon-inducible double stranded RNA dependent activator pseudogene 1	−1.927
ENSSSCG00000006612	S100A10	S100 calcium binding protein A10	−1.937
ENSSSCG00000007982	MPG	N-methylpurine-DNA glycosylase	−1.949
ENSSSCG00000015570	IVNS1ABP	Influenza virus NS1A binding protein	−1.963
ENSSSCG00000014170	CAST	Calpastatin	−2.067
ENSSSCG00000006661	VPS45	Vacuolar protein sorting 45 homolog (S. cerevisiae)	−2.105
ENSSSCG00000000125	LGALS1	Lectin, galactoside-binding, soluble, 1	−2.133
ENSSSCG00000009565	GAS6	Growth arrest-specific 6	−2.193
ENSSSCG00000008428	MSH2	MutS homolog 2	−2.509
ENSSSCG00000013252	F2	Prothrombin Precursor (EC 3.4.21.5) (Coagulation factor II)	−2.746
ENSSSCG00000005512	C5	Complement component 5	−2.793
ENSSSCG00000017473	TOP2A	Topoisomerase (DNA) II alpha 170 kDa	−4.029
ENSSSCG00000009330	ALOX5AP	Arachidonate 5-lipoxygenase-activating protein	−4.058
